# Effectiveness of auriculotherapy on anxiety during labor: did the authors switch outcomes or salami slice their trial?

**DOI:** 10.1590/1518-8345.4697.3381

**Published:** 2020-10-05

**Authors:** Richard Gray, Bridgina Mackay, Amanda Waters, Ellie Brown

**Affiliations:** 1 La Trobe University, College of Science Health and Engineering, Melbourne, Victoria, Australia.; 2 University of Melbourne, Centre for Youth Mental Health, Melbourne, Victoria, Australia.

**Letter to the Editor regarding the article “Mafetoni RR, Rodrigues MH, Jacob LMS, Shimo AKK. Effectiveness of auriculotherapy on anxiety during labor: a randomized clinical trial. Rev. Latino-Am. Enfermagem. 2018;26:e3030. DOI: http://dx.doi.org/10.1590/1518-8345.2471.3030.”**

Dear editors, we read with interest the paper published in Revista Latino-Americana de Enfermagem (RLAE) reporting the findings of a randomised controlled trial testing the effectiveness of auriculotherapy on anxiety^( 1 )^. We are writing to seek clarification from the study authors about the reporting of trial outcomes.

The trial by Mafetoni, et al.^( 1 )^ was prospectively registered with the Brazilian Registry of Clinical Trials before the trial first participant was recruited^( 2 )^. According to the registry entry (RBR-47hhbj), the authors aimed to recruit 99 women in labor, and the primary outcome was pain determined using a 0-10 visual analogue scale. However, as far as we can determine, pain data are not reported in this manuscript. The apparent discrepancy between registry entry and manuscript is concerning and may be indicative of selective outcome reporting – where the registered primary outcome is changed or omitted – by the authors^( 3 )^. The primary purposes of trial registration is to eliminate outcome switching because of the potential for this practice to distort the evidence base^( 4 )^. However, it requires that reviewers and editors reconcile what the authors have stated in the registry entry with what they report in the manuscript. Alternatively, the omission of pain data from the paper may be indicative of an intent to report outcomes across multiple papers. We investigated this possibility by reviewing the first authors SCOPUS entry (author ID “Mafetoni, Reginaldo Roque” 56560075300). According to SCOPUS, Mafetoni has published five papers, all reporting results from auriculotherapy trials. We checked the trial registration numbers reported in the five manuscripts. Three papers related to the trial registration reference RBR-47hhbj^( 1 , 5 - 6 )^. The first paper^( 6 )^ reports what are described as preliminary data from a three-arm (auriculotherapy, placebo, control) double-blind trial. Results from 30 participants (10 in each trial arm) are presented. The authors conclude that participants in the auriculotherapy group tended greater pain control. The authors also state that the “trial precedes a larger study in progress” [sic]^( 6 )^. The second manuscript^( 1 )^ is the trial published in RLAE that reports the effectiveness of auriculotherapy against anxiety in a triple-blind, three-arm, RCT involving 102 participants. It is unclear if the subjects in the first paper^( 6 )^ are part of 102 participants included in the second paper^( 1 )^. We wrote to the corresponding author on the 18^th^ June 2020 seeking clarification (they had not responded as of the 13^th^ July 2020). The authors report the effects of auriculotherapy against pain in a third paper^( 5 )^. Again, the trial is described as triple-blind and involved 102 participants. The authors conclude that auriculotherapy is an effective treatment in women in labor. Reporting of the results of a single study across multiple manuscripts – typically described as salami slicing^( 7 )^ – is a questionable research practice because of the potential to distort the evidence (for example, because papers from a single trial get included multiple times in systematic reviews). We would welcome an explanation from the authors about their approach to reporting their findings. It would also be informative if editors can check if reviewers or handling editor reconciled the submitted manuscript with the registry entry. If this was not done, can the editors explain why not?
