# Supplementary material for: Effectiveness of auriculotherapy on anxiety during labor: did the authors switch outcomes or salami slice their trial?
Source: Rev Lat Am Enfermagem. 2020 Oct 5;28:e3381. doi: 10.1590/1518-8345.4697.3381 (PMC7538062; doi:10.1590/1518-8345.4697.3381)
Supplement: Related texts

# Reply: Response of the authors of the article “Mafetoni RR, Rodrigues MH, Jacob LMS, Shimo AKK. Effectiveness of auriculotherapy on anxiety during labor: a randomized clinical trial. Rev. Latino-Am. Enfermagem. 2018;26:e3030. DOI: http://dx.doi.org/10.1590/1518-8345.2471.3030.”

**Authors:** Reginaldo Roque Mafetoni, Mariana Haddad Rodrigues, Lia Maristela da Silva Jacob, Antonieta Keiko Kakuda Shimo

**Affiliations:** 1 Universidade Paulista, Instituto de Ciências da Saúde, Campinas, SP, Brasil.; 2 Universidade Estadual de Londrina, Departamento de Enfermagem, Londrina, PR, Brasil.; 3 Universidade Estadual de Campinas, Faculdade de Enfermagem, Campinas, SP, Brasil.; 4 Centro Universitário de Jaguariúna, Faculdade de Jaguariúna, Jaguariúna, SP, Brasil.

Dear Researchers, we appreciate the interest in our publication and the request for clarifications, which we received with great esteem and consideration.

The article “Effectiveness of auriculotherapy on anxiety during labor”^( 1 )^is part of a larger study that is a doctoral thesis called “Effects of auriculotherapy on labor”, registry entry: RBR-47hhbj, of January 7, 2015, last updated on June 7, 2015. The study was approved by the local Research Ethics Committee (n. 855.496/CAAE: 35671514.6.0000.5404). A pilot study with 30 participants was previously conducted, from April to June 2015, to validate the data collection instrument and identify possible corrections to the study. Primary data on **labor pain** [visual analog scale (VAS) graded from 0 to 10] were taken from the pilot study for publication^( 2 )^. In the pilot study, blinding was considered for the participants and for the evaluators that collected the data, however, this was not performed, being later corrected in other publications through triple-blinding. The other data and other outcomes studied in the pilot study: **anxiety** (Hamilton Anxiety Rating Scale - HAM-A), **time of labor and type of delivery** , were recorded in a database.

The sample size was estimated at 99 participants. There were no changes to the variables or to the other characteristics studied in the pilot, therefore the complete data of the first 30 participants were included in the final analysis, giving a total of 102 participants (three more, predicting losses) in June 2016.

Therefore, in order to allow a specific analysis of the outcomes and broad discussion with the literature, at the end of the study, the primary outcomes: **labor pain** ^( 3 )^and **anxiety** ^( 1 )^, and the secondary outcomes: **time of labor and type of delivery** ^( 4 )^were included in different publications, although reported in the main study (RBR-47hhbj).

Best regards,

The authors.

## References

[1] Mafetoni RR, Rodrigues MH, Jacob LMS, Shimo AKK (2018). Effectiveness of auriculotherapy on anxiety during labor: a
randomised clinical trial. Rev. Latino-Am. Enfermagem.

[2] Mafetoni RR, Shimo AKK (2016). Effects of auriculotherapy on labour pain: a randomised
clinical trial. Rev Esc Enferm USP.

[3] Mafetoni RR, Rodrigues MH, Silva FMB, Jacob LMS, Shimo AKK (2019). Effectiveness of auticular therapy on labor pain: a
randomised clinical trial. Texto Contexto Enferm.

[4] Mafetoni RR, Jacob LMS, Jorge HMF, Shimo AKK (2018). Effects of auriculotherapy on labor time and cesarean section
rate: randomized clinical trial. Rev Mineira Enferm.
